# The emerging role of microRNAs and long noncoding RNAs in drug resistance of hepatocellular carcinoma

**DOI:** 10.1186/s12943-019-1086-z

**Published:** 2019-10-25

**Authors:** Ling Wei, Xingwu Wang, Liyan Lv, Jibing Liu, Huaixin Xing, Yemei Song, Mengyu Xie, Tianshui Lei, Nasha Zhang, Ming Yang

**Affiliations:** 1 Shandong Provincial Key Laboratory of Radiation Oncology, Cancer Research Center, Shandong Cancer Hospital and Institute, Shandong First Medical University and Shandong Academy of Medical Sciences, Jinan, 250117 Shandong Province China; 2 Department of Radiation Oncology, Shandong Cancer Hospital and Institute, Shandong First Medical University and Shandong Academy of Medical Sciences, Jinan, 250117 Shandong Province China; 3 Department of Intervention Surgery, Shandong Cancer Hospital and Institute, Shandong First Medical University and Shandong Academy of Medical Sciences, Jinan, China

**Keywords:** Hepatocellular carcinoma, Drug resistance, Long non-coding RNA, microRNA

## Abstract

Hepatocellular carcinoma (HCC) is the fifth most common malignancy worldwide and the second most lethal human cancer. A portion of patients with advanced HCC can significantly benefit from treatments with sorafenib, adriamycin, 5-fluorouracil and platinum drugs. However, most HCC patients eventually develop drug resistance, resulting in a poor prognosis. The mechanisms involved in HCC drug resistance are complex and inconclusive. Human transcripts without protein-coding potential are known as noncoding RNAs (ncRNAs), including microRNAs (miRNAs), small nucleolar RNAs (snoRNAs), long noncoding RNAs (lncRNAs) and circular RNA (circRNA). Accumulated evidences demonstrate that several deregulated miRNAs and lncRNAs are important regulators in the development of HCC drug resistance which elucidates their potential clinical implications. In this review, we summarized the detailed mechanisms by which miRNAs and lncRNAs affect HCC drug resistance. Multiple tumor-specific miRNAs and lncRNAs may serve as novel therapeutic targets and prognostic biomarkers for HCC.

## Background

Hepatocellular carcinoma (HCC) is the fifth most prevalent malignancy worldwide, accounting for approximately 90 % of primary liver cancer, characterized by high mortality, recurrence, metastasis and poor prognosis [1]. Due to the lack of symptoms in the early disease stages, most patients were diagnosed at the advanced disease stages with metastasis and poor hepatic reserve, thus losing the opportunity of curative resection. In the last decade, improved drug therapy agents have significantly prolonged the survival of HCC patients with advanced diseases. The commonly used therapeutic regimens include sorafenib, adriamycin (ADM), 5-fluorouracil (5-FU), platinum-containing anti-cancer drugs, camptothecin and gemcitabine [2–4]. However, the acquisition of multi-drug resistance (MDR) to these agents is the Achilles’ Heel in clinical oncology, and may result in a poor prognosis. Intrinsic or acquired drug resistance is defined as the resistance of malignant cells to different structurally and functionally unrelated anticancer drugs. The mechanisms involved in HCC drug resistance are complex and include the increased expression of drug efflux transporters that recognize and pump out anticancer drugs out of tumor cells, redistribution of intracellular accumulation of agents, inactivation of apoptosis signaling pathways, enhanced DNA damage repair capacity, accelerated drug metabolism and activation of cancer stem cells (CSCs) [5–7]. Up to now, however, the precise mechanisms underlying HCC drug resistance remain to be investigated.

Human transcripts without protein-coding potential are known as noncoding RNAs (ncRNAs). According to the length and shape, ncRNAs can be subdivided into the following types: tiny/short ncRNAs, long ncRNAs (lncRNAs) larger than 200 nucleotides (nt) and circular RNA (circRNAs). There are various small ncRNAs, such as microRNAs (miRNAs), PIWI-interacting RNAs (piRNAs), small nucleolar RNAs (snoRNAs) and small nuclear RNAs (snRNAs) [8–16]. NcRNAs were once considered as the “dark matter” or “by-products” during gene transcription. It has been found that ncRNAs are involved in various cellular functions, including proliferation, apoptosis and the cell cycle progression [17, 18]. For instance, snoRNAs are essential for guidance of chemical modifications of other RNAs, whereas tRNAs and rRNAs are critical for protein translation. MiRNAs and lncRNAs play a vital role in regulating gene expression via their fine regulation at various levels, including transcription, translation and protein functions. Multiple miRNAs and lncRNAs not only can serve as biomarkers for diagnosis of multiple cancers, but also are involved in drug resistance [19–26]. Lots of ncRNAs have been found to be abnormally expressed in HCC and to be associated with the invasion, metastasis, drug resistance and radioresistance of HCC cells [19–26]. Among the abnormally expressed ncRNAs, multiple miRNAs and lncRNAs have been found to play essential roles in HCC drug resistance.

In this review, we systematically summarize the literatures on miRNAs and lncRNAs modulating HCC drug resistance as well as the underlying mechanisms, thereby providing insight into the role of ncRNAs as putative biomarkers and/or therapeutic targets of HCC in the future.

### MiRNAs and therapy resistance

Currently, drug therapy for HCC includes targeted therapy, chemotherapy and immunotherapy. Molecular targeted drugs include sorafenib, regorafenib, lenvatinib and tivantinib. Chemotherapeutic drugs commonly used in clinics are adriamycin, 5-FU, cisplatin, and oxaliplatin. As for immunotherapy for HCC, it is a novel management choice and principally includes immune checkpoint blockers/monoclonal antibodies against the programmed cell death protein 1 (PD-1), PD-1 ligand (PD-L1) and receptor cytotoxic T lymphocyte antigen-4 (CTLA-4), such as nivolumab, pembrolizumab, MED14736, ipilimumab and tremelimumab.

As a class of endogenous, small, single-stranded ncRNAs of 19–24 nucleotides (nt) in length, miRNAs could bind to the 3′-untranslated regions (3′-UTRs) of target messenger RNAs (mRNAs) and suppress their expression and/or prohibit the translation. miRNAs are commonly involved in cellular differentiation, proliferation, death, angiogenesis and metabolic stress responses [27–29]. Dysregulated miRNAs act as oncogenes or tumor suppressors in the development and progression of many cancers, including HCC [30–32]. Interestingly, significantly altered miRNA expression have been found in a variety of drug-resistant HCC cells compared to those in drug-sensitive cells, suggesting that prediction of efficacy of different drugs using various miRNAs may promote individualized HCC therapy [25, 33–36]. miRNAs can regulate immune checkpoint molecules expression in tumor microenvironment [37]. However, it is still unclear how miRNAs are involved in resistance to immune checkpoint blockers currently. Moreover, emerging evidences indicate that multiple miRNAs involved in sorafenib resistance. By contrast, few miRNAs have been found to be participated in resistance to other novel targeted drugs.

### MiRNAs and resistance to sorafenib

Sorafenib, an oral multikinase inhibitor, was initially demonstrated to suppress proliferation and angiogenesis by targeting BRAF, Raf-1, Flt3, VEGFR-2/3 and PDGFR-β. Subsequently, it was also indicated that sorafenib could target signaling pathways independent of Raf, especially pathways regulating apoptosis and cell cycle progression [38–40]. As an FDA-approved standard targeted therapy agent for HCC, sorafenib showed survival benefits in advanced HCC patients worldwide. However, most patients eventually progressed to drug resistance diseases. Currently, the mechanisms involved in sorafenib resistance include activation of EGFR, c-Jun and/or AKT, microenvironmental hypoxia, epithelial-mesenchymal transition (EMT), activation of CSCs, resistance to apoptosis, cell cycle dysregulation, autophagy, and the abnormal expression of miRNAs and lncRNAs [36, 41–48]. A variety of miRNAs have been reported to be involved in sorafenib resistant in HCC (Table 1).

**Table 1:** MiRNAs and sorafenib resistance in HCC

MiRNAs	Expression^1^	Pathway	Reference
miR-21	up-regulated	PTEN/Akt	[49]
miR-93	up-regulated	PTEN; CDKN1A	[50]
miR-216a /217	up-regulated	PTEN; SMAD7	[51]
miR-181a	up-regulated	RASSF1	[52]
miR-494	up-regulated	PTEN	[53]
		mTOR	[45]
miR-221	up-regulated	caspase-3	[46]
miR-122	down-regulated	IGF-1R	[54]
		IGF-1R; SRF; ADAM10	[55]
		PDK4	[56]
		SLC7a1	[57]
		GALNT10	[58]
miR-34a	down-regulated	Bcl-2	[59]
miR-27b	down-regulated	P53	[60]
let-7	down-regulated	BCL-XL	[61]
miR-193b	down-regulated	MCL1	[62, 63]
miR-486	down-regulated	CLDN10; CITRON	[64]
miR-367-3p	down-regulated	Androgen receptor	[65]
miR-338-3p	down-regulated	HIF-1α	[43]
miR-137	down-regulated	ANT2	[66]
miR-142-3p	down-regulated	ATG5/ATG16L1	[36]
miR-7	down-regulated	TYRO3	[67]

^1^miRNAs either up-regulated or down-regulated in sorafenib resistant hepatocellular carcinoma cells

Note: This table shows 18 miRNAs, their expression level and potential targets in sorafenib resistance of hepatocellular carcinoma

Several oncogenic miRNAs can promote sorafenib resistance [45, 49–53]. For example, miR-93, miR-216a and miR-217 could confer sorafenib resistance by targeting *cell cycle protein-dependent kinase 1A* (*CDKN1A*) and modulating apoptosis as well as the TGF-β signaling [50, 51]. Additionally, miR-181 can trigger sorafenib resistance by targeting and suppressing *Ras association domain family member 1* (*RASSF1*) [52]. Exogenous expression of miR-494 has been found to increase resistance of HCC to sorafenib via targeting *PTEN* and activating the mTOR signaling. In animal models, sorafenib combined with anti-miR-494 potentiated the sensitivity of HCC to sorafenib, suggesting that miR-494 is a possible therapeutic target of advanced HCC [45, 53]. It was also found that miR-221 potentiates sorafenib resistance by suppressing caspase-3 mediated apoptosis in vivo. Moreover, its expression in serum has been found to be significantly lower in sorafenib responders compared to non-responders, suggesting that miR-221 may be a candidate biomarker to predict responders to sorafenib [46].

Meanwhile, multiple tumor suppressor miRNAs can reverse sorafenib resistance in HCC. MiR-122, for instance, has been found to be the most highly expressed miRNA in normal liver and remarkably reduced in sorafenib-resistant HCC cells. MiR-122 could promote sorafenib sensitivity of HCC cells through targeting and suppressing *insulin-like growth factor 1 receptor (IGF-1R)*, *serum response factor* (*SRF*), *depolymerization and metalloproteinase domain-containing protein 10* (*ADAM10*), *pyruvate dehydrogenase kinase 4* (*PDK4*), *solute carrier family 7 member 1* (*SLC7A1*) and *peptide N-acetyl N-acetylgalactosaminyl transferase 10* (*GALNT10*) [54–58]. Similarly, miR-34a has been found to reverse the tolerance of HCC cells to sorafenib via silencing *BCL-2* [59]. MiR-27b, let-7 and miR-193b which are curial regulators of apoptosis, also have been demonstrated to enhance sensitivity of HCC cells to sorafenib by silencing *P53*, *Bcl-XL* and/or *myeloid leukemia cell differentiation protein* (*MCL1*), respectively [60–63]. In addition, the tumor suppressor miR-486 and miR-367-3p have been shown to promote sorafenib sensitivity by inhibiting *CITRON*, *claudin 10* (*CLDN10*) and *androgen receptor* (*AR*) [64, 65]. It has been reported that miR-338-3p can sensitize HCC cells to sorafenib by silencing *hypoxia inducing factor 1α* (*HIF-1α*) in vitro and in vivo [43]. MiR-137 has been found to be significantly down-regulated in sorafenib-resistant Huh7-R HCC cells. Exogenous miR-137 can promote sorafenib sensitivity and inhibit cancer initiation cell (CIC) phenotype through targeting *adenine nucleotide transporter 2* (*ANT2*) [66]. Ectopic expression of miR-142-3p, a novel autophagy regulator, can sensitize HCC cells to sorafenib by silencing *autophagy related 5* (*ATG5*) and *autophagy related 16-like 1* (*ATG16L1*) and, thus, promote autophagy induced by sorafenib [36]. In addition, the tumor suppressor miR-7 has been found to promote sorafenib sensitivity in HCC cells through silencing expression of *TYRO3*, a member of TYRO3-AXL-MER family of receptor tyrosine kinases [67].

### MiRNAs and resistance to adriamycin

Adriamycin, an anthracycline antibiotic and non-specific periodic drug, is a strong inhibitor of DNA and RNA synthesis in tumor cells during proliferation. Adriamycin could diffuse into the nucleus of HCC cells, interact with DNA and eventually induce apoptosis. There are some known mechanisms for development of adriamycin resistance of HCC [68–75]. Multiple miRNAs have been reported to be involved in adriamycin resistance in HCC (Table 2).

**Table 2:** MiRNAs and adriamycin resistance in HCC

MiRNAs	Expression^1^	Pathway	Reference
Let-7a	up-regulated	Caspase–3	[76]
miR-519d	up-regulated	CDKN1A/p21, PTEN, AKT3, TIMP2	[77]
miR-26a/b	down-regulated	ULK1	[78]
miR-26b	down-regulated	TAK1, TAB3	[79]
miR-520b	down-regulated	ATG7	[80]
miR-491-3p	down-regulated	Sp3/ABCB1	[81]
miR-122	down-regulated	MDR1	[82]
		ABCB1; ABCF2	[83]
		PKM2	[84]
miR-31	down-regulated	NDRG3	[85]
miR-223	down-regulated	ABCB1	[86]
miR-133a,miR-326	down-regulated	ABCC1	[87]
miR-101	down-regulated	EZH2	[88]
		Mcl-1	[89]
miR-199a-3p	down-regulated	mTOR, c-Met	[90]
miR-215	down-regulated	DHFR, TS	[91]
miR-145	down-regulated	Smad3	[92]
miR-503	down-regulated	MDR1, MRP, ERCC1, Bcl-2	[93]

^1^miRNAs either up-regulated or down-regulated in adriamycin resistant hepatocellular carcinoma cells

Note: This table shows 17 miRNAs, their expression level and potential targets in adriamycin resistance of hepatocellular carcinoma

Several oncogenic miRNAs can promote adriamycin resistance. Let-7a has been found to increase resistance of HepG2 cells to adriamycin [76]. Exogenous expression of miR-519d has also been found to increase resistance of HCC cells to adriamycin by targeting multiple tumor suppressor genes, including *p21* and *PTEN* [77].

By contrast, it has been found that many tumor suppressor miRNAs can reverse adriamycin resistance in HCC. MiR-26a/b, for instance, has been found to be significantly down-regulated in 30 HCC tissues compared to normal tissues. In addition, exogenous miR-26a/b expression has been found to promote adriamycin sensitivity of HCC cells by targeting *ULK1* expression as well as the autophagy signaling pathway in vivo and in vitro [78]. Moreover, miR-26b can also sensitize HCC cells to adriamycin through silencing the TAK1 and TAB3 pathways [79]. Similarly, miR-520b has been found to increase the sensitivity of BEL-7402/ADM HCC cells to adriamycin via silencing expression of *ATG7*, a key autophagy regulator [80]. In HCC cells, expression of miR-491-3p are inversely associated with expression of *ABCB1* or *Sp3*. Consistently, ectopic expression of miR-491-3p could sensitize HCC cells to adriamycin by silencing expression of *ABCB1* or *Sp3* [81]. MiR-122, a highly expressed liver-specific miRNA in normal liver tissue, was significantly down-regulated in HCC. The tumor suppressor miR-122 has been found to promote adriamycin sensitivity in HCC cells through inhibiting cell cycle, anti-apoptotic effector factors and ABC transporters [82–84]. Similarly, miR-31 has been found to increase the sensitivity of HCC cells to adriamycin via silencing the expression of *NDRG3* [85]. In addition, down-regulated expression of tumor suppressor miRNAs, such as miR-223, miR-133a, miR-326, miR-101, miR-199a-3p, miR-215, miR-145 and miR-503, are significantly correlated with adriamycin resistance of HCC through inhibiting expression of the MDR-related genes. On the contrary, restoring the expression of these miRNAs could reverse adriamycin resistance of HCC cells [86–93].

### MiRNAs and resistance to 5-FU

5-FU, a heterocyclic aromatic chemotherapeutic agent, is a broadly used chemotherapeutic drug for HCC treatment. Through inhibiting thymidylate synthase (TS), 5-FU can interfere DNA replication and, thus, result in cell cycle arrest and apoptosis in response to DNA damage [94, 95]. Multiple oncogenic or tumor suppressive miRNAs have been found to be involved in 5-FU resistance (Table 3).

**Table 3:** MiRNAs and 5-FU resistance in HCC

MiRNAs	Expression^1^	Pathway	Reference
miR-200a-3p	up-regulated	DUSP6	[96]
miR-183	up-regulated	IDH2/SOCS6-HIF-1α	[97]
miR-141	up-regulated	Keap1	[98]
miR-193a-3p	up-regulated	SRSF2	[99]
miR-195	down-regulated	Bcl-w	[100]
miR-125b	down-regulated	Hexokinase II	[101]
Let-7 g	down-regulated	HMGA2	[102]
miR-133a, miR-326	down-regulated	Bcl-xl	[103]
miR-503	down-regulated	EIF4E	[104]

^1^miRNAs either up-regulated or down-regulated in resistant hepatocellular carcinoma cells

Note: This table shows 10 miRNAs, their expression level and potential targets in 5-FU resistance of hepatocellular carcinoma

It has been found that several oncogenic miRNAs can promote 5-FU resistance in HCC cells, including miR-200a-3p, miR-183, miR-141 and miR-193a-3p. For example, exogenous expression of miR-200a-3p enhanced 5-FU resistance of HCC cells by silencing *dual specificity phosphatase 6* (*DUSP6*) [96]. There was significantly higher miR-183 expression in 5-FU resistant HCC cells compared to 5-FU sensitive HCC cells. Knockdown of oncogenic miR-183 might significantly reverse the 5-FU tolerance [97]. It has been reported that miR-141 can promote the resistance to 5-FU in HCC cells by inhibiting *kelche-like ECH 1-related protein 1* (*Keap1*) and activating the antioxidant pathway [98]. Moreover, miR-193a-3p has been shown to enhance 5-FU resistance of HCC cells via suppressing *serine/arginine-rich splicing factor 2* (*SRSF2*) [99].

On the contrary, several tumor suppressor miRNAs may be associated with 5-FU resistance. Accumulated evidences indicate that upregulated *BCL-2* expression confers 5-FU resistance in HCC. It has been found that miR-195 could inhibit expression of *BCL-2*, and an increased sensitivity of drug-resistant HCC cells to 5-FU has been observed after exogenous overexpression of miR-195 [100]. Ectopic expression of miR-125b has been found to lead to reduced *hexokinase 2* (*HK2*) protein expression and to sensitize HCC cells to 5-FU by inhibiting glycolysis [101]. 5-FU resistant HCC cells have been found to exhibit reduced expression levels of let-7 g and exogenous expression of let-7 g could significantly sensitize HCC cells to 5-FU [102]. Similarly, miR-133a and miR-326 may restore chemosensitivity of HCC to 5-FU by targeting *Bcl-xl* [103]. MiR-503, which has been found to be significantly down-regulated in HCC tissues, could lead to significantly increased 5-FU toxicity of HCC cells by suppressing *eukaryotic translation initiation factor 4E* (*EIF4E*) [104].

### MiRNAs and resistance to cisplatin

Cisplatin, the first-generation of the platinum chemotherapeutic drugs, can inhibit DNA replication and transcription by forming crosslinks between DNA double strands and exhibits broad-spectrum antitumor activity. Cisplatin is one of the most commonly used chemotherapeutic agents to treat advanced HCC. Several miRNAs have been reported to be involved in cisplatin resistance in HCC (Table 4).

**Table 4:** MiRNAs and cisplatin resistance in HCC

MiRNA	Expression^1^	Pathway	Reference
miR-130a	up-regulated	RUNX3	[105]
miR-182	up-regulated	TP53INP1	[106]
miR-340	down-regulated	Nrf2	[107]
miR-363	down-regulated	Mcl-1	[108]

^1^miRNAs either up-regulated or down-regulated in cisplatin resistant hepatocellular carcinoma cells

Note: This table shows 4 miRNAs, their expression level and potential targets in cisplatin resistance of hepatocellular carcinoma

Multiple oncogenic miRNAs can promote cisplatin resistance, such as miR-130a and miR-182. Significantly elevated expression of miR-130a and miR-182 has been observed in tumor tissues from HCC patients and cisplatin-resistant Huh7 and HepG2 cells [105, 106]. Exogenous expression of miR-130a led to cisplatin tolerance of Huh7 cells by targeting the tumor suppressor *RUNX3* and activating the Wnt/β-catenin pathway [105]. Similarly, inhibition of miR-182 may partially conquer cisplatin resistance in cisplatin-resistant HepG2 cells by inhibiting tumor suppressor *tumor protein 53-induced nucleoprotein 1* (*TP53INP1*) [106].

By contrast, multiple tumor suppressor miRNAs can reverse cisplatin resistance of HCC. MiR-340, for instance, has been found to be significantly down-regulated in cisplatin-resistant HCC cells. In addition, exogenous miR-340 expression has been found to sensitize chemo-resistant HepG2/CDDP cells to cisplatin through silencing *Nrf2* expression, as well as the antioxidant pathway [107]. Additionally, miR-363 has been found to be significantly down-regulated in cisplatin resistant HepG2 cells compared to parental cells, and exogenous miR-363 could significantly reverse cisplatin tolerance in HepG2 cells by targeting *Mcl-1* [108].

### MiRNAs and resistance to other drugs

Paclitaxel, cetuximab and etoposide are also commonly used to treat HCC [109–111]. As shown in Table 5, several miRNAs are involved in resistance to these drugs. The tumor suppressor miR-16 has been found to be down-regulated in HCC tissues and to sensitize HCC cells to paclitaxel by suppressing the expression of *IKBKB* as well as the NF-κB signaling in vitro and in vivo [35]. Similarly, tumor suppressor miR-9 and miR-23a could sensitize HCC to cetuximab and etoposide by inhibiting expression of *eukaryotic translation initiation factor 5A2* (*eIF-5A-2*) and *topoisomerase 1* (*TOP1*), respectively [112, 113]. As a potential antitumor protein, tumor necrosis factor-related apoptosis inducing ligand (TRAIL) could selectively eliminate various types of HCC cells without exerting toxic effects in normal tissues. MiR-26b and miR-138 have been found to be involved in TRAIL-induced apoptosis and anti-malignancy in HCC. MiR-26b could promote the cytotoxicity of TRAIL in HCC cells by inhibiting *Mcl-1* [114]. Similarly, miR-138 was significantly down-regulated in TRAIL resistant HCC cells compared to TRAIL sensitive HCC cells. Ectopic expression of miR-138 has been found to improve the sensitivity of HCC cells to TRAIL [115].

**Table 5:** MiRNAs and other drugs resistance in HCC

MiRNAs	Expression^1^	Pathway	Drug	Reference
miR-16	down-regulated	IKBKB	paclitaxel	[35]
miR-9	down-regulated	eIF-5A-2	cetuximab	[112]
miR-23a	down-regulated	Topoisomerase 1(TOP1)	etoposide	[113]
miR-26b	down-regulated	Mcl-1	TRAIL	[114]
miR-138	down-regulated	interferon stimulating gene 15	TRAIL	[115]
miR-93	up-regulated	PTEN; CDKN1A	tivantinib	[50]

^1^miRNAs either up-regulated or down-regulated in other chemo-drugs resistant hepatocellular carcinoma cells

Note: This table shows 6 miRNAs, their expression level and potential targets in chemoresistance of hepatocellular carcinoma

In addition, miRNAs are also involved in resistance to tivantinib and regorafenib, two novel targeted drugs. Tivantinib, a small, selective oral inhibitor of c-Met receptor tyrosine kinase, provides an option as a second-line treatment for advanced HCC patients who have failed or are intolerant to sorafenib. It has been found that oncogenic miR-93 could confer tivantinib resistance by targeting *PTEN* and *CDKN1A* [50]. Interestingly, it has been reported that nine plasma miRNAs, including upregulated plasma expression of miR-30a, miR-122, miR-125b, miR-200a, and miR-374b, diminished levels of miR-15b, miR-107, and miR-320b, as well as the absence of miR-645, can predict regorafenib survival of advanced HCC patients [116].

### LncRNAs and therapy resistance

LncRNAs are a new class of ncRNAs longer than 200 nt and have no protein coding potential. Based on the positions and characteristics in human genome, lncRNAs can be divided into five categories: sense, antisense, bidirectional, intronic and intergenic lncRNAs [117–120]. Multiple lncRNAs are aberrantly expressed in HCC and are significantly associated with metastasis, recurrence, prognosis and chemoresistance through various mechanisms, including interactions with DNA, RNA or proteins to form complexes that regulate the expression of target genes [118, 121–130]. Several lncRNAs have been found to be involved in drug resistance in HCC (Table 6).

**Table 6:** LncRNAs and drug resistance in HCC

LncRNAs	Expression^1^	Pathway	Drugs	Reference
NR2F1-AS1	up-regulated	miR-363-ABCC1	oxaliplatin	[131]
HANR	up-regulated	GSKIP/P-GSK3β	adriamycin	[132]
lncARSR	up-regulated	PTEN-PI3K/Akt	adriamycin	[134]
HULC	up-regulated	USP22/Sirt1/ autophagy	oxaliplatin;5-FU; pirarubicin	[135]
MALAT1	up-regulated	miR-216b/ autophagy	5-FU; adriamycin;mitomycin	[136]
TUC338	up-regulated	RASAL1	sorafenib	[137]
VLDLR	up-regulated	ABCG2	sorafenib	[138]

^1^lncRNAs either up-regulated or down-regulated in chemo-resistant hepatocellular carcinoma cells

Note: This table shows 7 lncRNAs, their expression level and underlying pathways in the chemoresistance of hepatocellular carcinoma

It has been reported that lncRNA NR2F1-AS1 is significantly up-regulated in oxaliplatin-resistant HCC tissues and cell lines and confer HCC resistance to oxaliplatin. NR2F1-AS1 can promote tumor progression in vitro and in vivo. ABCC1 protein is a member of the superfamily of ATP-binding cassette (ABC) transporters which is involved in multi-drug resistance. In HCC cells, lncRNA NR2F1-AS1 could induce *ABCC1* expression via endogenous sponging miR-363 and, thus, antagonize chemosensitivity to oxaliplatin [131].

LncRNA HANR (HCC associated long non-coding RNA) shows evidently increased expression in HCC tissues and is associated with poor prognosis of HCC patients. Silencing of lncRNA HANR suppresses HCC proliferation in vitro and in vivo, enhances apoptosis and promotes sensitivity to doxorubicin. It has been found lncRNA HANR could bind to GSKIP and suppress the phosphorylation of GSK3β. In hepatocarcinogenesis, suppression of GSK3β phosphorylation and enhanced GSK3β total protein expression regulate glycogen metabolism and cell growth. As a result, alteration of endogenous cellular HANR expression influenced the sensitivity of HCC to doxorubicin-mediated chemotherapy [132].

LncARSR (lncRNA activated in renal cell carcinoma with sunitinib resistance) was firstly reported to be correlated with clinically poor sunitinib response in renal cell carcinoma. It has been found that lncARSR promotes sunitinib resistance via competitively binding miR-34/miR-449 to facilitate AXL and c-MET expression in renal cell carcinoma cells [133]. Recently, LncARSR is also found to play a part in doxorubicin resistance of HCC. LncARSR is significantly upregulated in HCC, associates with large tumor size and advanced disease stage. Overexpression of lncARSR promotes doxorubicin resistance of HCC cells in vitro and in vivo. In HCC cells, lncARSR physically associates with *PTEN* mRNA, enhances *PTEN* mRNA degradation, reduces *PTEN* expression, and activates the PI3K/Akt signaling pathway [134].

It has been found that lncRNA HULC (highly upregulated lncRNA in HCC) could induce autophagy through silencing expression of *silent information regulator 1* (*Sirt1*) protein and weaken the chemosensitivity of oxaliplatin, 5-FU and pirarubicin (THP) in HCC cells. It was also indicated that lncRNA HULC could up-regulate expression of *ubiquitin-specific peptidase 22* (*USP22*) and reduce the ubiquitin-mediated degradation of Sirt1 protein by removing the conjugated polyubiquitin chain of Sirt1 [135]. In addition, lncRNA MALAT1 has been found to be dramatically increased in 5-FU resistant HCC cells of BEL-7402/5-FU and can modulate MDR through impacting autophagy. Knockdown of MALAT1 can reverse 5-FU, adriamycin and mitomycin resistance, diminish LC3-II level and potentiate 5-FU induced apoptosis, which are similar with the effects of autophagy inhibitor of 3-Methyladenine (3-MA) [136].

Therapy resistance to targeted therapy for HCC, i.e. sorafenib, is one of major hurdles in clinics. LncRNA TUC338, for instance, has been found to be involved in the HCC sorafenib resistance. Elevated levels of lncRNA TUC338 were found both in HCC tissues and cell lines. Knockdown of lncRNA TUC338 sensitized HCC cells to sorafenib, which was accompanied by increased expression of *RASAL1* [137]. Similarly, lncRNA VLDLR is also involved in sorafenib resistance of HCC cells. VLDLR is one of lncRNAs contained within extracellular vesicles (EV) during chemotherapeutic stress in human HCC. Silencing VLDLR led to the decreased cell viability and altered cell cycle distribution, accompanied with the reduced expression of drug-resistant protein *ABCG2* (*ATP-binding cassette, subfamily G member 2*). Overexpression of ABCG2 protein reversed the effects of VLDLR knockdown on sorafenib-induced cell death [138].

There are several novel lncRNAs have been indicated to play crucial functions in HCC oxaliplatin resistance. Yin et al. evaluated the difference of genome-wide lncRNA expression profiling between oxaliplatin-sensitive and oxaliplatin-resistant HCC cells. A total of 120 differentially expressed lncRNAs were identified, among which 61 lncRNAs were up-regulated and 59 lncRNAs were down-regulated (fold changes > 2, *P* < 0.05). It was found that ENST00000502804, NR_073453 and ENST00000438347 were also up-regulated in drug-resistant HCC tissues and high expression of ENST00000438347 and ENST00000518376 was associated with bad outcomes of HCC patients [139].

## Conclusions and perspectives

**Fig. 1:**
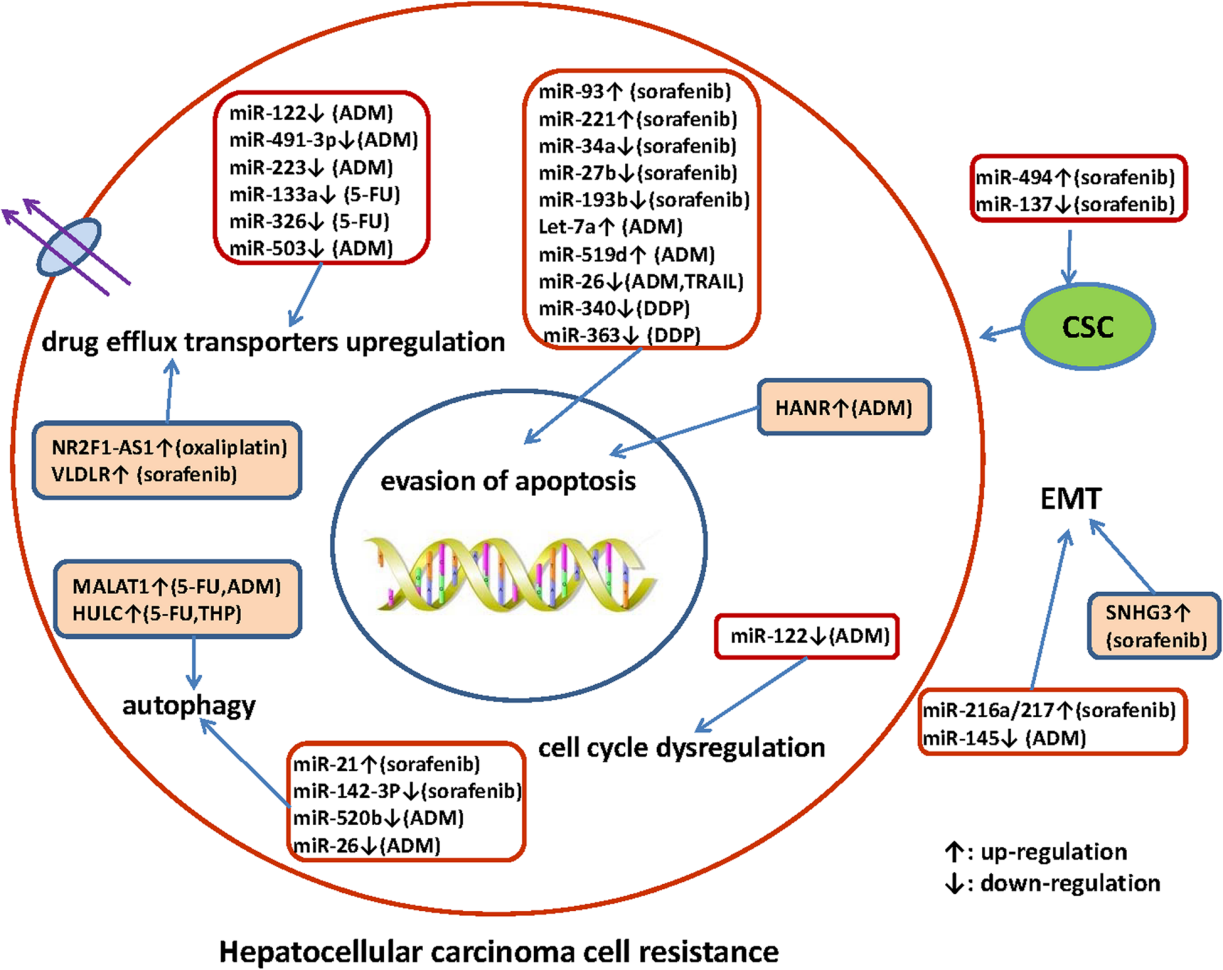
A summary diagram of miRNAs and lncRNAs involved in the drug resistance of hepatocellular carcinoma (HCC). Various miRNAs and lncRNAs could participate in drug resistance of HCC by affecting cell apoptosis, proliferation, cell cycle, autophagy, epithelial-mesenchymal transition, and cancer stem cell via modulating the expression of downstream target genes

More and more ncRNAs have been identified to be associated with chemoresistance in HCC. The mechanisms underlying the role of ncRNAs in HCC drug resistance are summarized in Fig. 1. Targeting these dysregulated endogenous miRNAs and/or lncRNAs may be a promising way to reverse drug resistance. For example, nanoparticle delivery of synthetic oligonucleotides targeting oncogenic miRNAs or synthetic tumor suppressor miRNAs or administration with natural agents for the regulation of miRNAs have been investigated as a proof-of-concept for HCC treatment. For lncRNAs, it is applicable that the direct delivery of tumor suppressive lncRNAs molecules to the target cells or the knockdown of oncogenic lncRNAs through specific siRNAs or shRNAs against them. Targeting ncRNAs in combination with conventional chemotherapeutics against HCC may be a promising alternative for reversing drug resistance and contributing to a better outcome in advanced HCC patients. However, it remains challenging to select critical target ncRNAs from numerous candidates. In order to promote ncRNA-based therapeutic interventions that benefit HCC patients, further investigations on additional translation research and clinical trials are urgently needed, which may ultimately open up potential approaches for overcoming HCC drug resistance.

## Data Availability

Not applicable.

## Abbreviations

3′-UTR: 3′-untranslated region
3-MA: 3-Methyladenine
5-FU: 5-fluorouracil
ABCG2: ATP-binding cassette, subfamily G member 2
ADAM10: Depolymerization and metalloproteinase domain-containing protein 10
ANT2: Adenine nucleotide transporter 2
AR: Androgen receptor
ATG16L1: Autophagy related 16-like 1
ATG5: Autophagy related 5
CDKN1A: Cell cycle protein-dependent kinase 1A
CIC: Cancer initiation cell
circRNA: circular RNA
CSC: Cancer stem cells
DUSP6: Dual specificity phosphatase 6
EIF4E: Eukaryotic translation initiation factor 4E
eIF-5A-2: Eukaryotic translation initiation factor 5A2
EMT: Epithelial-mesenchymal transition
GALNT10: Peptide N-acetyl N-acetylgalactosaminyl transferase 10
HCC: Hepatocellular carcinoma
HIF-1α: Hypoxia inducing factor 1α
HK2: Hexokinase 2
IGF-1R: Insulin-like growth factor 1 receptor
Keap1: Kelche-like ECH 1-related protein 1
lncRNA: Long noncoding RNA
MDR: Multi-drug resistance
miRNA/miR: microRNA
mRNA: messenger RNA
ncRNA: noncoding RNA
nt: nucleotide
PDK4: Pyruvate dehydrogenase kinase 4
piRNA: PIWI-interacting RNA
RASSF1: Ras association domain family member 1
rRNA: Ribosomal RNA
shRNA: Short hairpin RNA
siRNA: Small interfering RNA
Sirt1: Silent information regulator 1
SLC7A1: Solute carrier family 7 member 1
snoRNA: Small nucleolar RNA
SRF: Serum response factor
SRSF2: Serine/arginine-rich splicing factor 2
THP: Pirarubicin
TOP1: Topoisomerase 1
TP53INP1: Tumor protein 53-induced nucleoprotein 1
TRAIL: Tumor necrosis factor-related apoptosis inducing ligand
tRNA: Transfer RNA
TS: Thymidylate synthase
USP22: Ubiquitin-specific peptidase 22
